# Hemodialysis is Not Associated With Pre-prosthetic Inpatient Rehabilitation Outcomes After Dysvascular Lower Extremity Amputation: A Retrospective Cohort Study

**DOI:** 10.33137/cpoj.v3i2.34471

**Published:** 2020-09-04

**Authors:** W.S. Journeay, M.G. Marquez, M. Kowgier

**Affiliations:** 1 Providence Healthcare – Unity Health Toronto, Toronto, ON, Canada.; 2 Division of Physical Medicine and Rehabilitation, Department of Medicine, University of Toronto, Toronto, Canada.; 3 Department of Anatomy and Cell Biology, McGill University, Montreal, Canada.; 4 Dalla Lana School of Public Health, University of Toronto, Toronto, Canada.

**Keywords:** Dysvascular amputation, End-stage renal disease, Hemodialysis, Inpatient rehabilitation, Charlson comorbidity index

## Abstract

**BACKGROUND:**

Lower extremity amputation due to complications from peripheral vascular disease and/or diabetes are common and these patients often have multiple comorbidities. Patients with end-stage renal disease receiving hemodialysis (ESRD/HD) are a particularly vulnerable group at risk for amputation. After lower extremity amputation (LEA) surgery, many patients undergo post-operative inpatient rehabilitation to improve their pre-prosthetic functional independence. Given the increased complexity of dysvascular patients living with ESRD/HD compared to those without ESRD/HD, the association of HD with pre-prosthetic inpatient functional outcomes warrants further study.

**OBJECTIVE:**

The objective of this study was to compare the pre-prosthetic functional outcomes and Length of Stay (LOS) among patients with recent dysvascular LEA with and without ESRD/HD.

**METHODOLOGY:**

A retrospective cohort design was used to analyze a group of 167 patients with unilateral, dysvascular limb loss who were admitted to inpatient rehabilitation with 24 of these patients in the ESRD/HD group. Age, gender, amputation level, amputation side, length of stay (LOS), time since surgery, Functional Independence Measure (FIM) scores (admission and discharge), and Charlson Comorbidity Index (CCI) were collected.

**FINDINGS:**

There was no difference between patients with dysvascular amputation with and without ESRD/HD in the association of functional outcomes or LOS in this cohort and rehabilitation model. The CCI score was higher in the ESRD/HD group. Multivariate analysis indicated an inverse relationship with age and FIM scores, where increased age was associated with lower Total and Motor FIM at admission and discharge. There were no associations with FIM change. Age was positively associated with LOS. Being female was inversely associated to motor FIM scores at admission and discharge

**CONCLUSION:**

Among patients with recent dysvascular LEA, ESRD/HD is not associated with different functional outcomes or LOS in the pre-prosthetic inpatient rehabilitation setting. This suggests that despite added comorbidity that patients with ESRD/HD may still benefit from inpatient rehabilitation to optimize pre-prosthetic function.

## INTRODUCTION

Diabetes and peripheral arterial disease along with associated dysvascular complications are major risk factors for lower extremity amputation (LEA).^1–4^ Peripheral arterial disease and diabetes are associated with greater than 80% of LEA in Canada^4,5^ and recent data published by Hussain et al.,^6^ concluded that diabetes-related amputations are on the rise. Patients with comorbid diabetes and end-stage renal disease (ESRD) receiving hemodialysis (HD) are at particularly high risk of LEA.^7–9^

Individuals with dysvascular limb loss that also receive HD often have additional comorbidity, mortality and poorer ambulatory outcomes.^8,10–14^ Other common comorbidities in patients living with dysvascular amputation include hypertension, diabetes, heart disease and peripheral arterial disease. Data on the specific relationships between comorbidity and inpatient rehabilitation functional outcomes after LEA are still emerging^15,16^ with relatively less published on the rehabilitation of patients needing HD.^11,17^ Patients receiving HD tend to be medically complex and need to attend HD while also undergoing rehabilitation, which can potentially impact the course of an inpatient rehabilitation admission after LEA. Moreover, patients with ESRD tend to have more advanced comorbid disease and frailty^18^ and therefore LEA is often a significant medical event requiring post-operative rehabilitation.^19^ Given the poor long-term outcomes of patients with dysvascular amputation receiving HD^7,10,12,20^ it remains important to determine the postoperative, inpatient functional outcomes and rehabilitations needs as many patients will need to achieve a functional status sufficient to return home.^21^

Inpatient rehabilitation after LEA is particularly beneficial as it is associated with fewer additional amputations, reduced mortality, a greater probability of receiving a prosthesis, and improved medical stability.^22–24^ Regardless of one’s prosthetic candidacy, patients undergoing amputation have a number of post-operative rehabilitation needs including wound care, transfer training, wheelchair skills and contracture prevention which are needed to facilitate a safe discharge from a rehabilitation hospital. One commonly used measure of functional change in the inpatient rehabilitation setting is the Functional Independence Measure (FIM).^25,26^

Previous work by Arneja et al.^11^ demonstrated that patients with recent limb loss who were receiving HD had a longer length of stay (LOS) in rehabilitation and lower FIM scores than patients who did not require HD. This retrospective study included prosthetic rehabilitation outcomes with FIM scores reported at discharge from inpatient rehabilitation and at mean follow up of 13.8 months (3-31 months). They did not include a standardized index of comorbidity or admission FIM scores. Czyrny & Merrill^27^ also compared 19 patients with LEA with ESRD receiving HD and 19 patients with peripheral vascular disease-related amputation not receiving HD. They studied ambulatory outcomes in addition to FIM at admission, discharge and FIM change. They noted an increased burden of comorbidity in the ESRD group but found no differences between the two groups in functional outcomes at discharge which included ambulation with a prosthesis.

We aimed to compare the pre-prosthetic functional outcomes and LOS among patients with dysvascular LEA with and without ESRD/HD. This work adds to the literature by using the Charlson Comorbidity Index (CCI) in this population and capturing both the admission and discharge FIM for pre-prosthetic, post-operative inpatient rehabilitation in patients with recent LEA.

## METHODOLOGY

This was a retrospective cohort study and was approved by the Research Ethics Board of Providence Healthcare and closed by the Unity Health Toronto Research Ethics Board. All patients with a LEA that were discharged from our rehabilitation hospital between January 1, 2014 and March 30, 2018 were identified and their medical records were reviewed. Inclusion criteria for the study consisted of those with a recent unilateral, transfemoral (TF) or transtibial (TT) amputation. Only patients with amputations due to a dysvascular etiology were included, and those due to trauma, cancer, or other reasons were excluded. Those patients receiving HD who also met the inclusion criteria were included. Inclusion and exclusion criteria were developed to establish a uniform data set of the most common reason for admission to post-amputation inpatient rehabilitation (dysvascular amputation). Patients who met inclusion criteria but had an incomplete data set were excluded. All data retrieved from medical records came from both physical charts and electronic files utilized by Health Information Management at the hospital.

The rehabilitation model at this institution involved postoperative interdisciplinary rehabilitation including physiotherapy, occupational therapy, nursing, wound care, and physiatry consultation. Patients did not receive HD on-site but were able to travel to their HD treatments at outside facilities three days per week. The focus of rehabilitation for these patients was solely pre-prosthetic rehabilitation which includes, but is not limited to: wound care, standing tolerance, contracture prevention, ADLs, transfers, and wheelchair skills. Patients were discharged home after pre-prosthetic rehabilitation and then were revisited regarding prosthetic candidacy and gait training at a later date.

Data that was extracted from the medical records included age, sex, amputation level, amputation side, surgery date, LOS in inpatient rehabilitation, FIM scores at admission and discharge,^25,26^ and CCI total score.^28,29^ The authors are aware that the CCI was initially used as an epidemiological tool to predict mortality in patients admitted to hospital. However, we have selected the CCI as a standardized method in which to catalogue comorbidities and have used it in previous published work.^15^ Each patient was reviewed using the CCI and assigned points for the individual conditions, then given a total score. These scores were based on information present upon their admission and any past medical history that was documented in the chart. The time since surgery was also recorded by calculating the number of days between the surgery date and the admission date to inpatient rehabilitation. LOS in rehabilitation was calculated from admission date to discharge date. Total FIM, and total motor FIM, information was retrieved from admission and discharge data. We included motor FIM because in the pre-prosthetic phase of rehabilitation the motor FIM scores would reflect acquisition of independence with transfers and wheelchair mobility as this study did not examine prosthetic gait outcomes.

### Statistical Methods

Continuous variables were summarized by observed means with standard deviation (SD) and categorical variables were summarized by frequency counts (percentages). Univariate and multivariate linear regression analyses were used to investigate the effect of HD on each of the outcomes of Total and Motor FIM, at both admission and discharge separately (i.e., cross-sectional effects), as well as LOS. To investigate the longitudinal effects, changes between discharge and admission were computed for both Total (FIM Total change) and Motor FIM (Motor FIM Change). Univariate and multivariate linear regression analyses were used to investigate the effect of HD on each of the outcomes of FIM Total Change and Motor FIM Change. Multiple regression analysis adjusted for clinically relevant variables including age, sex, amputation level, amputation side, and the Charlson Comorbidity Index. Data was analyzed using the R statistical software (version 3.5.1).

## RESULTS

All patients admitted with a diagnosis of LEA from January 1, 2014 to March 30, 2018 were identified by our medical records team for a total of 382 records. Three patients were excluded due to death prior to discharge. Four patients were excluded due to incomplete admission to discharge data sets. Two hundred and eight patients were excluded by not meeting inclusion criteria such as: etiology of amputation (i.e. not dysvascular), had bilateral amputations, or were not TT or TF level amputations (i.e. only forefoot or toe amputation), or were not admitted post-operatively but rather for other reasons such as gait training or other medical conditions. There was a total of 167 patients with dysvascular amputation meeting the inclusion criteria with 24 of these patients receiving HD (Table 1).

**Table 1:** Cohort description. *P<0.05.

		**Dysvascular Cohort**	
		**No Hemodialysis** **n=143**	**Hemodialysis** **n=24**
**Age (years)**		67.7 (SD=11.1)	64 (SD=7.4)
**Sex**	*M*	95(66%)	18(75%)
	*F*	48(33%)	6(25%)
**Amputation**	*Transfemoral*	59 (41%)	9 (38%)
**Level**	*Transtibial*	84 (59%)	15 (62%)
**Amputation**	*Left*	69 (48)	12 (50%)
**Side**	*Right*	74 (52)	12 (50%)
**Time since surgery to admission (days)**		15.2 (SD=13.8)	17.3 (SD=10.6)
**Length of stay in rehabilitation (days)**		33.9 (SD=18.6)	32.4 (SD=17.2)
**Charlson Comorbidity Index**		4.7 (SD=1.7)	8.0 (SD=1.7)*
**FIM scores**	*Total* *Admission*	72.6 (SD=14.4)	73.2 (SD=13.5)
	*Total* *Discharge*	97.5 (SD=14.3)	97.2 (SD=11.1)
	*Motor Total Admission*	42.7 (SD=12.0)	42.3 (SD=12.6)
	*Motor Total Discharge*	66.9 (SD=11.4)	65.8 (SD=9.5)
	*Efficiency*	0.9 (SD=0.5)	0.9 (SD=0.6)

A descriptive comparison of the dysvascular and dysvascular with ESRD/HD groups showed a significant difference between the CCI scores [4.7 (SD=1.7) vs 8.0 (SD=1.7)], P<0.001. Table 1 presents further descriptive data, and a demographic comparison of the dysvascular only and HD groups.

After univariate analysis, age was negatively associated with both Total FIM at admission (Beta -0.58, CI [ (-0.78) - (-0.39)], P<0.001) and at discharge (Beta -0.48, CI [ (-0.66) - (-0.29)] P<0.001). Age was also associated with motor FIM at admission (Beta -0.44, CI [ (-0.60) - (-0.28)] P<0.001) and at discharge (Beta -0.34, CI [ (-0.49) - (-0.19)] P<0.001). Sex was also associated with motor FIM at admission (Beta -4.13, CI [ (-7.99) - (-0.27)], P=0.038) and discharge (Beta -4.28, CI [ (-7.83) - (-0.73)], P=0.019). Age showed a relationship with LOS that was nearly statistically significant in the univariate analysis (Beta 0.25, CI [ (-0.01) - (0.50)], P=0.066) and was therefore carried forward in the multivariate analysis. There were no associations between the HD vs no HD. The remaining univariate analyses are presented in Table 2.

**Table 2:** **A:** Univariate Analysis – FIM Total Admission and Discharge, FIM Change, LOS, *P<0.05. **B:** Univariate analysis – FIM Motor Admission and Discharge, FIM Motor Change

	**FIM Total Admission**			**FIM Total Discharge**			**FIM Total Change**			**Length of Stay (LOS)**		
**A**	Beta	CI	P-value	Beta	CI	P-value	Beta	CI	P-value	Beta	CI	P-value
Sex, Female vs Male	-3.50	[(-8.10) - (1.1)]	0.138	-3.99	[(-8.46) - (0.47)]	0.081	-0.50	[(-378) - (279)]	0767	-0.68	[(-6.66) - (5.29)]	0.823
Amp Side, Left vs Right	-0.58	[(-4.91) - (375)]	0793	-2.19	[(-6.40) - (2.01)]	0.308	-1.61	[(-4.68) - (1.45)]	0.304	1.64	[(-3.94) - (7.23)]	0.565
Amp Level, TF vs TT	-2.63	[(-7.02) - (1.77)]	0.243	-2.28	[(-6.56) - (2.00)]	0.298	-0.35	[(-278) - (3.47)]	0.828	-3.35	[(-9.02) - (2.32)]	0.249
Age	-0.58	[(-078) - (-0.39)]	0*	-0.48	[(-0.66) - (-0.29)]	0*	-0.09	[(-0.05) - (-0.24)]	0.213	0.25	[(-0.01) - (0.50)]	0.066
HD vs no HD	-0.52	[(-670) - (5.65)]	0.868	0.30	[(-5.71) - (6.31)]	0.922	0.83	[(-3.55) - (5.20)]	0.712	1.49	[(-6.48) - (9.45)]	0.715
Charlson Comorbidity Index	-0.65	[(-1 70) - (0.40)]	0.227	-0.61	[(-1.63 - (0.41)]	0.245	0.04	[(-070) - (079)]	0.913	0.24	[(-1.11) - (1.60)]	0725
	**FIM Motor Admission**			**FIM Motor Discharge**			**FIM Motor Change**					
**B**	Beta	CI	P-value	Beta	CI	P-value	Beta	CI	P-value			
Sex, Female vs Male	-4.13	[(-7.99) - (-0.27)]	0.038*	-4.28	[(-7.83) - (-073)]	0.019*	-0.15	[(-3.21) - (2.91)]	0.924			
Amp Side, Left vs Right	0.57	[(-3.10) - (4.23)]	0762	-1.49	[(-4.86) - (1..88)]	0.388	-2.05	[(-4.90) - (0.80)]	0.160			
Amp Level, TF vs TT	-2.83	[(-6.09) - (1..33)]	0.210	-2.88	[(-6.29) - (0.53)]	0.100	-0.50	[(-3.42) - (2.42)]	0737			
Age	-0.44	[(-0.60) - (-0.28)]	0*	-0.34	[(-0.49) - (-0.19)]	0*	0.11	[(-0.03) - (0.24)]	0.120			
HD vs no HD	0.41	[(-4.80) - (5.63)]	0.876	1.19	[(-3.62) - (6.00)]	0.629	077	[(-3.31) - (4.86)]	0.711			
Charlson Comorbidity Index	-0.60	[(-1.49) - (0.28)]	0.183	-0.67	[(-1.49 - (0.14)]	0.107	-0.07	[(-077 - (0.63)]	0.847			

The factors that showed an association after the univariate analysis or were clinically relevant were then adjusted using multivariate analysis. Greater age was shown to be associated with lower Total FIM scores at admission (Estimate -0.59, SE 0.10, P<0.001) and discharge (Estimate -0.49, SE 0.10, P<0.001). Age was associated with motor FIM at admission (Estimate -0.46, SE 0.08, P<0.001) and discharge (Estimate -0.33, SE 0.08, P<0.001). Being female was inversely associated to motor FIM scores at admission (Estimate -4.50, SE 1.84, P=0.016) and discharge (Estimate -4.21, SE 1.75, P=0.017). Age was positively associated with LOS (Estimate 0.28, SE 0.14, P=0.044). Table 3 includes remaining data from multivariate analysis.

**Table 3:** **A:** Multivariate analysis – FIM Total Admission and Discharge, FIM Change, LOS, *P<0.05. **B:** Multivariate analysis – FIM Motor Admission and Discharge, FIM Change.

	**FIM Total Admission**			**FIM Total Discharge**			**FIM Total Change**			**Length of Stay (LOS)**		
**A**	Estimate (SE)	t-value	P-value	Estimate (SE)	t-value	P-value	Estimate (SE)	t-value	P-value	Estimate(SE)	t-value	P-value
Intercept	117.65 (8.07)	14.57	0	135.25 (8.07)	16.76	0.000	17.61 (6.38)	2.76	0.006	10.06 (11.49)	0.88	0.382
Sex, Female vs Male	-372 (2.17)	-1.71	0.088	-4.00 (2.17)	-1.85	0.067	-0.28 (1.71)	-0.16	0.870	-0.42 (3.08)	-0.14	0.892
Amp Side, Left vs Right	1.07 (2.03)	0.53	0.600	-0.72 (2.03)	-0.35	0.724	-1.79 (1.61)	-1.11	0.268	1.19 (2.89)	0.41	0.680
Amp Level, TF vs TT	0.86 (2.11)	0.41	0.685	0.69 (2.11)	0.33	0.743	-0.16 (1.67)	-0.10	0.922	-4.95 (3.00)	-1.65	0.100
Age	-0.59 (0.10)	-6.02	0*	-0.49 (0.10)	-4.99	0*	0.10 (0.08)	1.30	0.196	0.28 (0.14)	2.03	0.044*
HD vs no HD	-0.94 (3.51)	-0.27	0.790	-0.06 (3.51)	0.02	0.987	0.88 (2.78)	0.32	0.751	3.06 (5.00)	0.61	0.541
Charlson Comorbidity Index	-0.86 (0.60)	-1.43	0.154	-0.73 (0.60)	-1.22	0.224	0.13 (0.47)	0.27	0.789	0.71 (0.85)	0.83	0.407
	**FIM Motor Admission**			**FIM Motor Discharge**			**FIM Motor Change**					
**B**	Estimate (SE)	t-value	P-value	Estimate (SE)	t-value	P-value	Estimate (SE)	t-value	P-value			
Intercept	77.03 (6.86)	11.22	0	94.04 (6.51)	14.45	0	17.01 (5.91)	2.88	0.005			
Sex, Female vs Male	-4.50 (1..84)	-2.44	0.016*	-4.21 (1.75)	-2.41	0.017*	0.28 (1.59)	0.18	0.858			
Amp Side, Left vs Right	2.06 (1.73)	1.19	0.234	-0.34 (1..64)	-0.21	0.836	-2.40 (1.49)	-1.62	0.108			
Amp Level, TF vs TT	0.49 (1.79)	0.28	0.784	-0.64 (1.70)	-0.38	0.706	-1.14 (1.54)	-0.74	0.463			
Age	-0.46 (0.08)	-5.59	0*	-0.33 (0.08)	-4.25	0*	0.13 (0.07)	1.82	0.071			
HD vs no HD	0.35 (2.99)	0.12	0.906	0.50 (2.83)	0.18	0.859	0.15 (2.57)	0.06	0.954			
Charlson Comorbidity Index	-0.64 (0.51)	-1.27	0.206	-0.68 (0.48)	-1.42	0.158	-0.04 (0.44)	-0.09	0.932			

## DISCUSSION

The objective of this study was to examine the association of HD with pre-prosthetic inpatient rehabilitation outcomes and LOS in a cohort of patients with dysvascular LEA. Notable findings from this study included: **1.** Both groups of patients were similar in the amputation characteristics however patients with HD had a higher CCI indicating a greater burden of comorbidity **2.** Despite a higher CCI in the HD group there was no difference in FIM scores or LOS. **3.** Age and sex were associated with Total and motor FIM at admission and discharge and age was associated with LOS.

This study is unique in that it examines the CCI in patients with dysvascular limb loss with and without ESRD/HD and the association with inpatient pre-prosthetic functional outcomes and LOS. Prior work would suggest that patients living with ESRD/HD have greater medical complexity and an increased number of comorbidities.^11,14,20^ However, our data add to the literature by reporting the CCI in an inpatient rehabilitation cohort. Our previous work examined the CCI and its components in a dysvascular group of patients with limb loss but excluded patients receiving HD.^15^ Given the literature suggesting a number of poorer outcomes after LEA we sought to compare this group to patients without ESRD/HD in the inpatient rehabilitation setting. While the CCI was higher in the HD group, we found no associations with FIM or LOS. Although speculative, it is possible that increased comorbidity may be associated with poorer ambulatory outcomes, but is not associated with short-duration pre-prosthetic functional gains.

The literature would suggest that patients with dysvascular LEA who also have ESRD/HD have much higher mortality rates than those without HD and very low rates of ambulation with a prosthesis. One study reported that <10% of patients receiving HD retained their ability to ambulate at 1-year after amputation.^12^ It has also been shown that in patients with dysvascular LEA that those who are ambulatory have higher survival than those who do not ambulate.^12,14^ While inpatient rehabilitation has been shown to increase the likelihood of receiving a prosthesis in those with limb loss,^22^ it remains unknown whether this is true in the ESRD/HD population. Therefore, after amputation surgery these patients may still benefit from inpatient rehabilitation to maintain their pre-prosthetic independence. Specifically, in patients living with ESRD/HD where survival and ambulatory outcomes are poor, post-operative inpatient rehabilitation can allow for medical monitoring, transfer and wheelchair training and assessment of ADL prior to discharge from the inpatient rehabilitation hospital setting.^19^ Our data would suggest that despite greater comorbidity in patients with dysvascular amputation with ESRD/HD, there is no difference in Total and motor FIM or LOS compared to patients without ESRD/HD for pre-prosthetic inpatient rehabilitation.

A study by Arneja et al.^11^ examined functional outcomes of patients with LEA receiving HD and those without HD. In their study only discharge FIM scores were included, while our study contained both admission and discharge FIM. Additionally, their study examined various comorbidities but did not use an established index such as the CCI. Overall, in our study the total CCI score did not show association with pre-prosthetic functional outcomes in this cohort after multivariate analysis. FIM changes and scores in our cohort indicate the acquisition of independence with ADL, transfers and wheelchair mobility as this study did not examine prosthetic gait outcomes.

These patients are medically complex and admitted for pre-prosthetic rehabilitation, so their functional change as reflected by the FIM would be different than studies that have included ambulation as an outcome. Given the increased comorbidities and frailty in the ESRD/HD group, this data suggests that patients undergoing dysvascular amputation can still derive benefit from pre-prosthetic rehabilitation even if they are receiving HD. Additionally, there was no association of comorbidity with LOS suggesting that despite an increased burden of comorbidity and attendance at HD during rehabilitation, these patients can achieve a pre-prosthetic functional level that supports a safe discharge in a similar amount of time to non-ESRD/HD patients with recent LEA while admitted to inpatient rehabilitation.

Age was a factor that was found to have an association with total and motor FIM at both admission and discharge, with advanced age associated with lower FIM scores. There was not an association with FIM change, however, suggesting that despite lower FIM at admission and discharge the rate of change during the inpatient stay was not associated with age. There are prior studies that support the notion that advanced age is associated with poorer functional outcomes in patients with limb loss.^30,31^ However, another report by Chopra et al.^14^ did not indicate an association between greater age and poorer ambulatory rates, which they attributed to their cohort size. It is also well established that age is a powerful prognostic factor in gait retraining after amputation.^32^ In other work, age was also associated with decreased survival post amputation in patients receiving HD.^12^ Thus, while age was associated with FIM at the time of admission and discharge, this group of patients did derive benefit from pre-prosthetic inpatient rehabilitation regardless of their future prosthetic candidacy. Additionally, age was associated with LOS, suggesting that the older, dysvascular patient with recent LEA may require additional time in hospital to reach pre-prosthetic functional independence.

### Limitations

While prior reports indicated that ESRD/HD is associated with increased mortality and lower ambulatory function after dysvascular amputation,^12,14,33^ this cohort admitted for pre-prosthetic rehabilitation was not impacted. This suggests that patients with dysvascular LEA admitted postoperatively and who may never be prosthetic candidates may still benefit from inpatient rehabilitation to recover from surgery and restore independence prior to discharge even with ESRD/HD. The CCI reflects specific medical comorbidities however other factors may also play a role in rehabilitation after limb loss including the condition of the contralateral limb, visual impairments, delayed wound healing and mental health status, which could be explored in future studies. Furthermore, this cohort represents one post-amputation care model in Canada and therefore the results may not be directly generalized to other forms of rehabilitation services in different centers.

## CONCLUSION

We conducted this study to examine the role of ESRD/HD in pre-prosthetic inpatient rehabilitation functional outcomes and LOS in a cohort of dysvascular patients with recent lower extremity amputation. ESRD/HD was not associated with poorer FIM scores or LOS. In keeping with previously published work, we did find association with age and the admission and discharge total FIM, motor FIM and LOS. These data suggest that despite the medical complexity, higher mortality and poorer prognosis for ambulation after LEA in patients living with ESRD/HD, they have a similar pre-prosthetic, inpatient rehabilitation functional benefit as dysvascular patients who not have ESRD/HD.

## DECLARATION OF CONFLICTING INTERESTS

The authors have no conflicts of interest to declare.

## AUTHOR CONTRIBUTION

**W. Shane Journeay**: Conceived the study and design, data interpretation and led the manuscript writing.

**Michelle G. Marquez**: Data collection, data interpretation, literature review and assisted in manuscript writing.

**Matthew Kowgier**: Assisted in study design, led statistical analysis and contributed to manuscript development

## SOURCES OF SUPPORT

Michelle G. Marquez received a Providence Healthcare student research stipend.

## ETHICAL APPROVAL

This was a retrospective cohort study and was approved by the Research Ethics Board of Providence Healthcare and closed by the Unity Health Toronto Research Ethics Board.
